# The Effect of Ice Cream Intake on Pain Relief for Patients After Tonsillectomy

**DOI:** 10.7759/cureus.9092

**Published:** 2020-07-09

**Authors:** Majid A Albeladi, Marzouqi A Salamah, Rayan Alhussaini

**Affiliations:** 1 Otolaryngology-Head and Neck Surgery, Ohud Hospital, Al-Madinah Al-Munawarah, SAU; 2 Otolaryngology-Head and Neck Surgery, Prince Mohammed Bin Abdulaziz Hospital - National Guard Health Affairs, Al-Madinah, SAU

**Keywords:** ice cream, tonsillectomy, postoperative, pain, children

## Abstract

Introduction

The post-tonsillectomy pain can lead to a decrease in fluid and food intake, followed by dehydration, which can slow down the repair process and make pain control harder. Different groups of analgesics have their own side effects. Therefore, the consideration of non-pharmacological ways to control pain can be of great value such as ice cream and other cold drinks.

Aim

The purpose behind this study is to assess whether the use of ice cream after tonsillectomy with or without adenoidectomy in children reduces pain in the immediate postoperative period, as well as to compare the effect of ice cream and diet at room temperature on post-tonsillectomy pain in children.

Materials and methods

Each patient’s post-operative pain was evaluated and assessed by nursing staff prior to discharge using two reliable pain scale: FLACC scale (F: Face, L: Legs, A: Activity, C: Cry, C: Consolability) for patient less than seven years and Wong Baker pain scale (Face 0, very happy because he doesn’t hurt, Face 1, hurts a little bit, Face 2, hurts a little more, Face 3, hurts even more, Face 4, hurts a whole lot, Face 5, hurts as much as you can imagine, although you do not have to be crying to fell this bad) for patient more than seven years.

Results

The ice cream intake is significantly associated with having no pain (p-value 0.014). In univariate regression, compared to preschool, school-aged children have significant effect with the ice cream intake postoperatively (OR = 0.286, p-value 0.039) while sex and instrument used to assess pain score were having no significant effect with the ice cream intake postoperatively.

Conclusion

Further research is needed in order to validate the effectivity of ice cream intakes after tonsillectomy in our region.

## Introduction

The first removal of the tonsils was reported by the Greek physician Celsus about 2000 years ago. Celsus described his surgical procedure as scratching around the tonsils, releasing, and then removing them. He used vinegar and milk for hemostasis to reduce hemorrhage [1]. Tonsillectomy is still the most commonly performed surgical procedure for children in the United States with more than 530,000 procedures conducted annually. The most common indications of tonsillectomy are sleep-disordered breathing, tonsillitis, and peritonsillar abscess [1].

The most widely used technique to perform this operation is cold dissection method in which scalpel or surgical knife is utilized. After tonsillectomy, children should tolerate sore throats for one to two weeks and mostly experience referred pain in the ear. Acetaminophen is usually prescribed in order to control pain. The administration of steroids can also reduce pain and nausea [1].

Currently, a single dose of dexamethasone is recommended during the surgery. The post-tonsillectomy pain can lead to a decrease in fluid and food intake, followed by dehydration, which can slow down the repair process and make pain control harder. Different groups of analgesics have their own side effects. Therefore, the consideration of non-pharmacological ways to control pain can be of great value [1].

## Materials and methods

A total of 50 children within the age range of 3-15 years who underwent tonsillectomy were randomly assigned into two groups - group 1 with ice cream served to them and group 2 with a room-temperature diet served to them postoperatively. Each patient’s post-operative pain was evaluated and assessed by nursing staff prior to discharge using two reliable pain scale: FLACC scale (F: Face, L: Legs, A: Activity, C: Cry, C: Consolability) for patient less than seven years (Appendices), and Wong Baker pain scale (Face 0, very happy because he doesn’t hurt, Face 1, hurts a little bit, Face 2, hurts a little more, Face 3, hurts even more, Face 4, hurts a whole lot, Face 5, hurts as much as you can imagine, although you do not have to be crying to fell this bad) for patient more than seven years (Appendices) [2,3].

## Results

A total of 50 children underwent tonsillectomy were enrolled in this study. Table 1 presents the baseline characteristics of children. Age range was from 6 to 13 years (mean 6.3 ± 02.9). A high proportion of patients were boys (64.0%). Those who received ice cream postoperatively (case group) were 58% while those who did not receive ice cream (control group) were 42%. FLACC scale was the instrument used to measure pain for the preschool children (60%) whereas Wong Baker scale was the instrument used for school-aged children (40%). Only four cases had shown an indication of pain based on the instrument where almost all of the patients had shown oral intake tolerance (96%) whereas most of them discharged home from day case surgery (98%).

**Table 1:** Baseline characteristics of patients

Study data	N (%) (n = 50)
Age group in years	
Preschool (3 – 6 years)	30 (60.0%)
School-aged child (7 – 13 years)	20 (40.0%)
Sex	
Boy	32 (64.0%)
Girl	18 (36.0%)
Patients received ice cream post-operatively	
Yes	29 (58.0%)
No	21 (42.0%)
Instruments used to assess pain score	
FLACC scale (<7 years old)	30 (60.0%)
Wong Baker scale (≥7 years old)	20 (40.0%)
Level of Pain	
With pain	4 (8.0%)
No pain	46 (92.0%)
Patients oral intake tolerance	
Yes	48 (96.0%)
No	2 (4.0%)
Patients discharged home from day case surgery	
Yes	49 (98.0%)
No	1 (2.0%)

When measuring the association between ice cream intake postoperatively and the baseline characteristics of patients, we found that school-aged children were significantly more in the ice cream intake group (p-value 0.035). Ice cream intake was significantly associated with having no pain (p-value 0.014). The distribution of ice cream intakes was higher in boys compared to girls but no association was found (Table 2).

**Table 2:** Association between ice cream intake postoperatively and the baseline characteristics of patients (n = 50) ^§^ P-value has been calculated using chi square test. ** Significant at p ≤ 0.05 level.

Factor	Ice Cream Intake		P-value ^§^
	Yes, N (%) (n = 29)	No, N (%) (n = 21)	
Age group in years			
Preschool	21 (72.4%)	9 (42.9%)	0.035 **
School-aged child	8 (27.6%)	12 (57.1%)	
Sex			
Boy	19 (65.5%)	13 (61.9%)	0.793
Girl	10 (34.5%)	8 (38.1%)	
Instrument used to assess pain score			
FLACC scale	19 (65.5%)	11 (52.4%)	0.349
Wong Baker scale	10 (34.5%)	10 (47.6%)	
Level of pain			
With pain	0	4 (19.0%)	0.014 **
No pain	29 (100%)	17 (81.0%)	
Patient oral intake tolerance			
Yes	28 (96.6%)	20 (95.2%)	0.815
No	1 (3.4%)	1 (4.8%)	

In univariate regression, compared to preschool, school-aged children had significant effect with the ice cream intake postoperatively (OR = 0.286, p-value 0.039) while sex and instrument used to assess pain score had no significant effect with the ice cream intake postoperatively (Table 3).

**Table 3:** Binary regression analysis to predict the effect of ice cream intake from the baseline characteristics of patients (n = 50) CI – Confidence Interval. ** Significant at p ≤ 0.05 level.

Factor	Odds Ratio	95% CI	P-value
Age group in years			
Preschool	Ref		0.039 **
School-aged child	0.286	0.087 – 0.937	
Sex			
Boy	Ref		0.793
Girl	0.855	0.266 – 2.748	
Instrument used to assess pain score			
FLACC scale	Ref		0.351
Wong Baker scale	0.579	0.184 – 1.826	

## Discussion

Various forms of complication might happen during the course of surgery or right after tonsillectomy and the most commonly known among them was pain. Pharmacological agents such as analgesic have limited time of effects and might causes adverse effect. Children also complained of swallowing some agents that left a bad taste to their mouth. Different recommendations have been proposed for the management of pain. Some of them were: intravenous hydration, diet at room temperature, steroids or antibiotics and cold diet. The purpose behind this study is to assess whether the use of ice cream after tonsillectomy with or without adenoidectomy in children reduces pain in the immediate postoperative period. Furthermore, we want to compare the effect of ice cream and diet at room temperature on post-tonsillectomy pain in children. In this study, we randomly selected 58% patients for ice cream intake while for diet at room temperature 42% patients were selected. This selection criteria were also in accordance to the paper published by Meybodian et al. [1]. The study was about to measure the “effect of cold diet and diet at room temperature on post-tonsillectomy pain in children” where they divided the cases into two groups such as: 48.6% children were provided with cold-served diet (case group) and 51.4% were provided diet in room temperature (control group). A single-blinded, randomized controlled trial published in United Kingdom examined the use of ice-lollies for pain relief post-pediatric tonsillectomy [4]. They also divided 88 patients into two group such as: 47 cases allocated to receive an ice-lolly and 41 cases not to receive ice-lolly, which was also corroborated with our method. In South Korea, they divided patients into two groups equally where group 1 received cold-water cooling of the tonsillar fossa (20 cases) whereas group 2 did not receive cold-water cooling, which also supported our criteria [5]. This had also been indicated in a study published in Turkey, where they divided their cases into two group such as, group 1 as irrigated group and group 2 as control group [6].

In this study, we used the Face, Legs, Activity, Cry, Consolability (FLACC) scale to assess the pain of the pre-school group of patients. The scale is scored in a range of 0-10 with 0 representing no pain. The scale has five criteria, each of which are assigned a score of 0, 1 or 2 [2]. On the other hand, we utilized Wong-Baker Faces Pain Rating scale to measure the pain for school-aged children [3]. This is a pain scale that was developed by Donna Wong and Connie Baker. The scale shows a series of faces ranging from a happy face at 0, or "no hurt", to a crying face at 10, which represents "hurts like the worst pain imaginable". Based on the faces and written descriptions, the patient chooses the face that best describes their level of pain. FLACC pain scale had also been utilized in a study published by Meybodian et al. [1]. They measured the pain prior to oral diet initiation after the operation, before the second acetaminophen dose, before the next day breakfast and before discharge. Moreover, in UK, they used Modified Children’s Hospital of Eastern Ontario Pain Scale (Mcheops) to assess the pain of post-pediatric tonsillectomy [4]. The in-charge nursing staff assessed the pain of the patients at interval time of 15, 30, 60 minutes and 4 hours post-pediatric tonsillectomy while visual analogue scale (VAS) was the instrument employed to assess the pain of children by the papers published in Korea and Turkey [5-6].

Furthermore, when measuring the effectivity of ice cream intakes among children who underwent tonsillectomy, we found that ice cream intake is significantly associated with having no pain. In the developed countries such as UK, Korea and Turkey, they documented that the healing process was significantly improved upon the intakes of ice lollies, receiving of cold-water cooling and cooling the tonsillar fossae, and they measured the pain in a series of period of time [5-6]. On the other notes, in a paper published in the United States, they reported that the administration of cold things by mouth was the number one nonpharmacologic strategy for pain relief identified by 59.5% of children [7]. The use of popsicles was named specifically as an effective intervention. They further elaborated that 10.1% of the children used soft foods which resulted in decreasing of pain and 5.1% of them reported that the application of external cold packs improved pain relief. These findings supported that cold drinks are one of the best methods in the management of pain post-tonsillectomy.

## Conclusions

This study revealed that ice cream intake is significantly effective among children who underwent tonsillectomy. Further research is needed in order to validate the effectivity of ice cream intakes after tonsillectomy in our region.

## Figures and Tables

**Figure 1:**
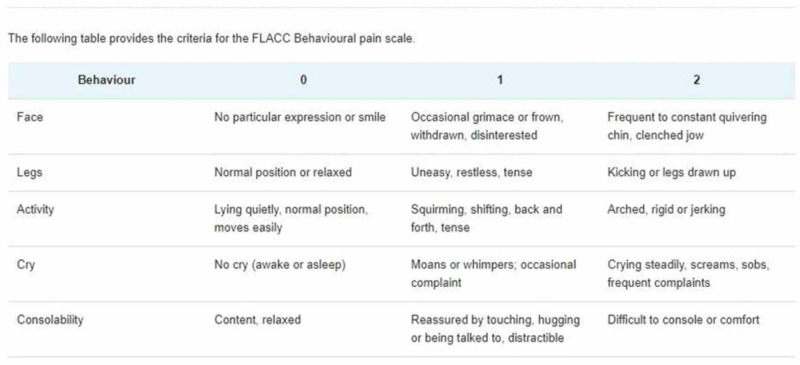
FLACC pain scale FLACC = F: Face, L: Legs, A: Activity, C: Cry, C: Consolability

**Figure 2:**
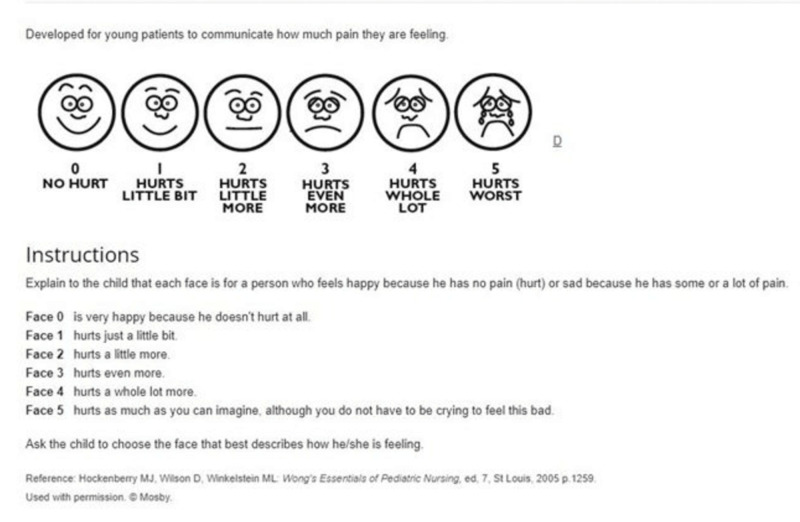
Wong Baker FACES pain rating scale
